# *De Novo* Venom-Gland Transcriptomics of Spine-Bellied Sea Snake (*Hydrophis curtus*) from Penang, Malaysia—Next-Generation Sequencing, Functional Annotation and Toxinological Correlation

**DOI:** 10.3390/toxins13020127

**Published:** 2021-02-09

**Authors:** Choo Hock Tan, Kae Yi Tan

**Affiliations:** 1 Venom Research and Toxicoogy Lab, Department of Pharmacology, Faculty of Medicine, University of Malaya, Kuala Lumpur 50603, Malaysia; 2 Protein and Interactomics Lab, Department of Molecular Medicine, Faculty of Medicine, University of Malaya, Kuala Lumpur 50603, Malaysia; kytan_kae@um.edu.my

**Keywords:** snakebite, envenomation, *Lapemis hardwickii*, *Hydrophis hardwickii*, three-finger toxin, alpha-neurotoxin

## Abstract

Envenomation resulted from sea snake bite is a highly lethal health hazard in Southeast Asia. Although commonly caused by sea snakes of Hydrophiinae, each species is evolutionarily distinct and thus, unveiling the toxin gene diversity within individual species is important. Applying next-generation sequencing, this study investigated the venom-gland transcriptome of *Hydrophis curtus* (spine-bellied sea snake) from Penang, West Malaysia. The transcriptome was *de novo* assembled, followed by gene annotation and sequence analyses. Transcripts with toxin annotation were only 96 in number but highly expressed, constituting 48.18% of total FPKM in the overall transcriptome. Of the 21 toxin families, three-finger toxins (3FTX) were the most abundantly expressed and functionally diverse, followed by phospholipases A_2_. Lh_FTX001 (short neurotoxin) and Lh_FTX013 (long neurotoxin) were the most dominant 3FTXs expressed, consistent with the pathophysiology of envenomation. Lh_FTX001 and Lh_FTX013 were variable in amino acid compositions and predicted epitopes, while Lh_FTX001 showed high sequence similarity with the short neurotoxin from *Hydrophis schistosus*, supporting cross-neutralization effect of Sea Snake Antivenom. Other toxins of low gene expression, for example, snake venom metalloproteinases and L-amino acid oxidases not commonly studied in sea snake venom were also identified, enriching the knowledgebase of sea snake toxins for future study.

## 1. Introduction

Snakebite envenomation is a World Health Organisation (WHO)-classified neglected tropical disease that heavily affects many impoverished populations in the tropics and subtropics [1]. Each year, it causes 81,000 to 138,000 deaths across the world and approximately three times as many permanent disabilities and psychological trauma in those who survive [2,3]. Snakebite cases are mostly reported from inland areas where agricultural activities are predominant, whereas sea snake bite remains an under-estimated, fatal occupational hazard to fishermen and coastal communities [4,5]. In recent years, the risk of sea snake bite increases due to various environmental and anthropogenic factors, for example, climate change (global warming) and economic practices such as sea snake hunting and trading, which are not uncommon in Asia [6,7].

Clinically, envenomation caused by sea snake bite is highly lethal, with a fatality rate between 3.2% and 30% [8]. The victims typically develop generalized neuromuscular paralysis, myotoxicity and complications such as acute kidney injury due to rhabdomyolysis [9,10]. The majority of studies on sea snake envenomation and pathophysiology were reported since early 1950s for the beaked sea snake (*Hydrophis schistosus* or *Enhydrina schistosa* prior to taxonomic revision [11]) in the western coast of Peninsular Malaya, ostensibly due to its common occurrence and frequent bites in the waters. More recent studies reported the venom proteomes of a few sea snake species, including the beaked sea (*H. schistosus*) and its congener species *Hydrophis cyanocinctus*, *Hydrophis curtus* and *Hydrophis (Pelamis) platura* [12,13,14,15,16], which represent the viviparous true sea snakes under the Hydrophiinae subfamily. The hydrophiids are relatively young radiations (approximately 3–5-million-year-old) comprising at least 16 genera, with the genera *Aipysurus* and *Hydrophis* being the two major clades [17,18]. Unlike *Aipysurus* which has a relatively stable taxonomic history and is mainly restricted in the Australo-Papuan region, the *Hydrophis* complex is a far more diverse monophyletic clade that consists of close to 50 species distributed in the waters of Indo-Malayan Archipelago, Indonesia, Australia and South China Sea. Each species of *Hydrophis* is evolutionarily distinct and thus, further genetic analysis is important to unveil the diversity and novelty of toxin genes within individual species.

In the current study, we investigated the venom gene profile of *H. curtus*, a medically and commercially important sea snake species in Southeast Asia through *de novo* venom-gland transcriptomics, applying next-generation sequencing technique. *Hydrophis curtus* (Synonym: *Hydrophis hardwickii*), commonly known as the spine-bellied sea snake, distributes widely from the Persian Gulf to the Indian coastline, Myanmar, Thailand, Straits of Malacca, Strait of Taiwan, South China Sea, the Philippines, Indonesia, Papua New Guinea and northern as well as eastern Australia [19]. In Southeast Asia, the increased human contact with sea snakes from various anthropogenic activities poses a threat to a wider community of people besides fishermen [20]. In this part of the world, sea snakes are a significant part of the global wildlife trade where they are captured, bred and harvested for live snake parts used in the production of accessories and for food (sea snake meat as an exotic delicacy), as well as for medicinal use (tonic soup, snake wine, gall bladder as traditional medicament) [7]. Admittedly, in the Gulf of Thailand, *H. curtus* is commonly implicated in the catch and trade industry of wild snakes and reported 80 tonnes of venomous sea snakes are harvested each year in the region. The economic benefit of sea snake trade to the fishers and traders clearly outweighs the risk of envenomation, while the snake population and the ecosystem are being jeopardized. To address the medical and ecological implications of sea snake toxins, the present study set to investigate the venom-gland transcriptomic profile of *H. curtus*, through which the toxin gene diversity of this species can be better understood and the existing venom database of sea snakes can be further enriched.

## 2. Results and Discussion

### 2.1. Sequencing and De Novo Transcriptome Assembly

**Figure 1:**
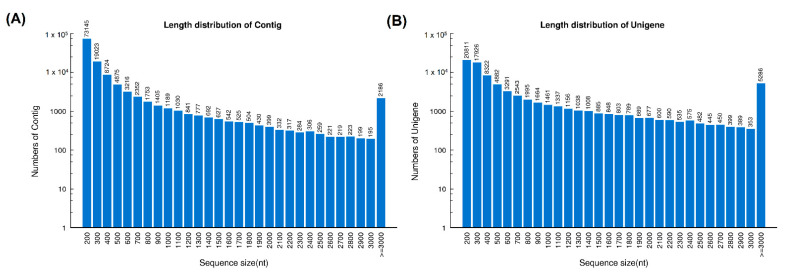
Length distribution of contigs (**A**) and unigenes (**B**) from the *de novo* assembly of *H. curtus* venom-gland transcriptome.

Sequencing of the cDNA libraries of *H. curtus* venom-gland tissue yielded a total of 54,140,326 clean reads. *De novo* assembly of the reads resulted in 126,790 contigs (N50 = 921), which were further clustered and streamlined into 82,209 unigenes (N50 = 2073). Of these, a total of 70,564 transcripts were identified at FPKM ≥ 1, a cut-off for gene expression of the current study (Table 1). The length distributions of contigs and unigenes from the *de novo* assembly of the venom-gland transcriptome were shown in Figure 1.

**Figure 2:**
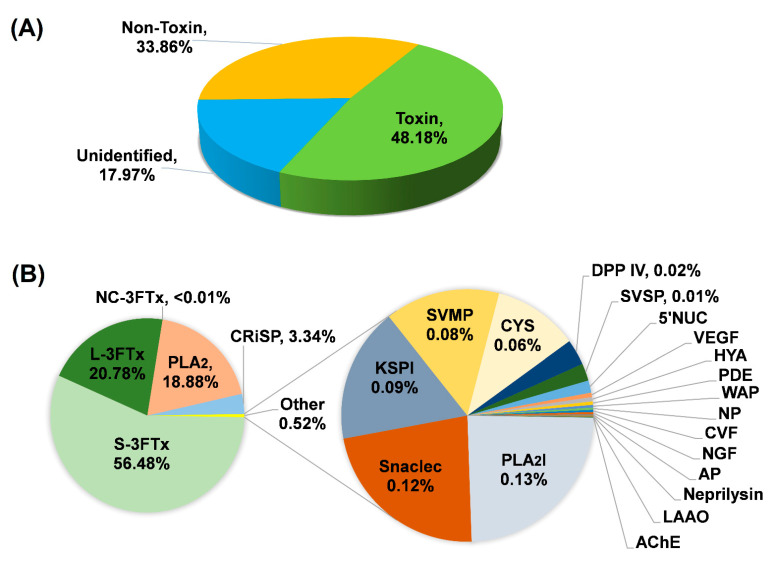
*De novo* transcriptome of *Hydrophis curtus* venom glands: (**A**) Overview profile of all-transcript expression. (**B**) Profiling of toxin transcripts by gene families. Abbreviations: S-3FTx, short three-finger toxin; L-3FTx, long three-finger toxin; NC-3FTx, non-conventional three-finger toxin; PLA_2_, phospholipase A_2_; CRiSP, cysteine-rich secretory protein; PLA_2_I, phospholipase A_2_ inhibitor; Snaclec, snake venom C-type lectin/lectin-like protein; KSPI, Kunitz-type serine protease inhibitor; SVMP, snake venom metalloproteinase; CYS, cystatin; DPP IV, dipeptidylpeptidase IV; SVSP, snake venom serine protease; 5′NUC, 5′ nucleotidase; VEGF, vascular endothelial growth factor; HYA, hyaluronidase; PDE, phosphodiesterase; WAP, waprin; NP, natriuretic peptide; CVF, cobra venom factor; NGF, nerve growth factor; AP, aminopeptidase; LAAO, L-amino acid oxidase; and AChE, acetylcholinesterase. The expression of 5′NUC, VEGF, HYA, PDE, WAP, NP, CVF, NGF, AP, neprilysin, LAAO and AChE were each <0.01% of total toxin FPKM.

Based on BLASTx search, the transcripts were assigned into three categories: (a) “Toxin”; (b) “Non-toxin”; and (c) “Unidentified” (Figure 2A; Table 1). Transcripts in the “Toxin” category encoded known and putative snake toxins; these constituted the venom-gland transcriptome by a total FPKM of 48.18%. The remaining portion of the transcriptome was shared between “Non-toxin” transcripts, which represent cellular or house-keeping genes (33.86% of the total FPKM) and transcripts with no identifiable hits from the BLASTx search (17.97% of total FPKM). The venom-gland transcriptome of *H. curtus* was dominated (virtually 50%) by transcripts with toxin annotation, reflective of the specialized toxin-secreting function of the gland tissue. The high expression of “genes for toxins” is comparable to previous findings in other terrestrial elapid snakes, including cobras (*Naja* spp.) [21,22], king cobra (*Ophiophagus hannah*) [23] and *Micrurus* spp. (American coral snakes) [24,25], where toxin transcripts accounted for more than 40% of the venom-gland transcriptomes.

### 2.2. Toxin Gene Expression Profile 

A total of 70,564 transcripts were expressed in the *H. curtus* venom-gland, while only 96 were classified under the “Toxin” category. The transcripts showed distinct, non-redundant sequences and were clustered by sequence similarity into 21 families of toxin genes (Figure 2B). Despite their extremely small number (96 out of 70,564 or 0.14% by total transcript count), these toxin transcripts were highly expressed, together contributing to an exceptionally high redundancy value of 4847.54 FPKM/transcript, which is in sharp contrast to the remaining genes with low expressions (non-toxin: 13.16 FPKM/transcript; unidentified: 3.89 FPKM/transcript, respectively) (Table 1). The high redundancy of toxin genes probably reflects multigene duplication in snake venom evolution, in which functional diversity of toxins with increasing prey-specificity is generated. By natural selection, this is essential for the evolving snakes to become more adapted to survive environmental perturbations and to occupy new niches for survival [26,27].

### 2.3. Profiling of Toxin Transcripts

The *de novo* transcriptome of *H. curtus* venom-gland also revealed high sequence similarity of all toxin transcripts to sequences from various elapid species (Table 2). Amongst the 96 toxin genes identified, 45 have full-length sequences with ≥90% amino acid coverage (Table 3). These proteins with full-length sequences included previously uncharacterized families of CRISP, CTL, KSPI, SVMP, cystatin, SVSP, 5NT, VEGF, hyaluronidase, waprin, CVF and neprilysin from sea snake venoms, in addition to the well-established 3FTx and PLA_2_ proteins. The *de novo* sequences of these toxins, which are unique to *H. curtus* are now available in the public repository database and provided in Supplementary Table S1 for deeper insights in the diversity of sea snake toxins. 

Of the 21 toxin gene families, 3FTxs are the most diversified and abundantly expressed (18 transcripts, 77.26% of total toxin FPKM), supporting that 3FTXs play a major role in the predatory function of *H. curtus* venom. More specifically, it was the alpha-neurotoxins that formed the bulk of 3FTx in the venom (~77% of total toxin FPKM) (Table 2). The diversely expressed 3FTx genes were further categorized into long-chain, short-chain and non-conventional groups (Table 2) and elaborated based on their functional attributes in the context of envenomation. PLA_2_ proteins constituted the second most abundantly expressed toxin genes (18.88% of total toxin FPKM), followed by CRISP (3.34%), PLA_2_ inhibitor (0.13%), CTL (0.12%), KSPI (0.09%), SVMP (0.087%), cystatin (0.06%), dipeptidyl peptidase (0.02%), SVSP (0.01%) and miscellaneous (5NT, VEGF, hyaluronidase, PDE, waprin, NP, CVF, NGF, aminopeptidase, neprilysin, LAAO and AChE, at <0.01% of total toxin FPKM, respectively). The transcriptomic profile showed a greater toxin diversity compared to the proteomic profile of *H. curtus* venom from the same geographical source (Penang) as reported previously [13], in which a few minor families (SVSP, NP, CTL, peptidases, neprilysin, AChE, waprin) were undetected at the protein level, implying that these minor proteins were present at a very low abundance in the venom but potentially serve ancillary function. From the evolutionary perspective, the transcriptomic finding indicates that the genes are conserved in the *Hydrophis* lineage, while their ecological significance awaits further elucidation.

### 2.4. Sequence Analysis and Phylogenetics of Three-Finger Toxins

Three-finger toxins are non-enzymatic polypeptides containing 60–74 amino acid residues orientated in three beta-stranded loops, resembling three protruding fingers [28,29]. Based on the protein structure, we categorized the 19 3FTx transcripts in *H. curtus* venom-gland transcriptome into short-chain 3FTx (S-3FTx, with four disulfide bridges), long-chain 3FTx (L-3FTx, with an additional fifth disulfide bridge on the second loop) and non-conventional 3FTx (NC-3FTx, with an additional fifth disulfide bridge on the first loop) [29]. The majority of the transcripts that contributed to 56.48% of total toxin FPKM, were found to be S-3FTX (10 transcripts). The L-3FTX, comprising 7 transcripts, constituted 20.78% of total toxin FPKM, while there was only one NC-3FX transcript present at a negligible abundance (<0.01%) (Table 2).

**Figure 3:**
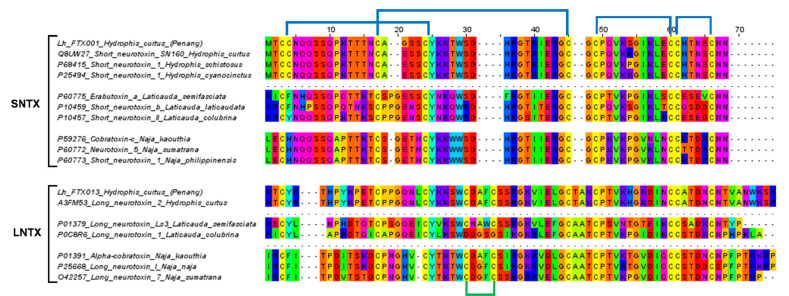
Multiple sequence alignment of the major short neurotoxins (SNTX) and long neurotoxins (LNTX) from *Hydrophis curtus* (Penang) and related representative species of sea snakes, sea kraits and cobras. All SNTX shared highly conserved cysteine residues and four disulphide bridges that reinforce the three-finger structure of the molecule. LNTX has an additional fifth disulphide bond in the second loop, except Ls3 from *Laticauda colubrina*.

Within S-3FTX and L-3FTX subgroups, the short neurotoxin transcript, Lh_FTX01 and the long neurotoxin transcript, Lh_FTX13 were, respectively, the most abundantly expressed transcripts. Lh_FTX01 was most similar to the short neurotoxin SN160 (UniProt: Q8UW27), previously cloned from *Lapemis hardwickii* (Guangxi, China) [30]. Both Lh_FTX13 and SN160 (Q8UW27) encoded proteins consisting of 60 amino acid residues but with minor variation at two residual positions: Gly^19^ in Lh_FTX01 was substituted by Glu^19^ in SN160 and Ser^46^ in Lh_FTX_01 was substituted by Arg^46^ in SN160, as shown in Figure 3. The variation observed could be due to genetic differences between distant geographical populations, as the present specimen was from the northern waters of Malacca Straits (coastal Penang Island) while the previous specimen was sourced from Beihai, Guangxi, Southern China. In comparison, high homology was observed amongst the short neurotoxin (SNTX) sequences of congeneric sea snakes (*Hydrophis* spp.), sea kraits (*Laticauda* spp.) and Asiatic cobras (*Naja* spp.), all of which are polypeptides of 60 amino acid residues reinforced by 4 disulfide bridges (8 highly conserved cysteine residues) (Figure 3). The sequence of Lh_FTX13, on the other hand, matched identically to the long neurotoxin 2 (UniProt: A3FM53), which was cloned from the same Chinese specimen. Lh_FTX013 showed conserved cysteine residues and disulfide bridges as with the long neurotoxin sequences of other comparing elapid species, although long neurotoxin (LNTX) sequences, in general, were more variable in amino acid composition. Notably, the LNTX of *L. colubrina* (P0C8R6) has only four disulfide bridges instead of five [31]. Comparing to Lh_FTX013 and other related LNTX sequences, P0C8R6 lacks the additional fifth disulfide bridge with amino acid mutation at residue-26 (C→D) and residue-30 (C→G), although the mutation did not appear to compromise its neurotoxicity and lethality [32]. 

**Figure 4:**
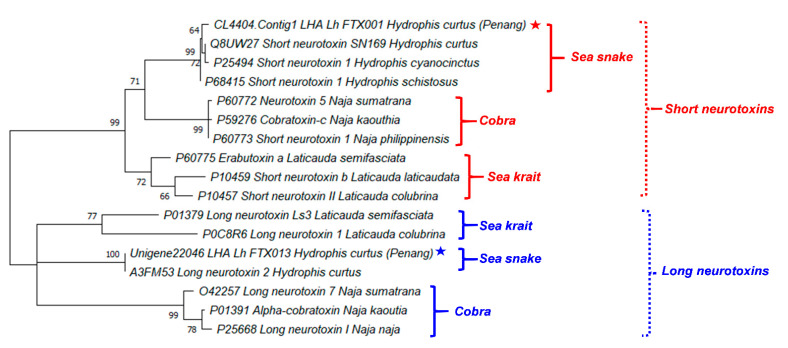
A phylogenetic tree of short and long neurotoxins from representative species of sea snakes, sea kraits and cobras. Stars indicated the major short and long neurotoxins derived from *de novo* venom-gland transcriptome of *Hydrophis curtus*, Penang. Tree was constructed with PAM model of Dayhoff and bootstrapping was performed with 1000 replicates on MEGA Version X [35]. Numbers indicate branch support values. Red/blue stars indicate the specimen studied in this work.

Figure 4 illustrates the phylogenetic tree of the major neurotoxins from *H. curtus* (Penang) and representative species of sea snakes, sea kraits and cobras. The SNTX and LNTX groups formed two distinct paraphyletic clades. The SNTX of sea snakes (*Hydrophis* spp.) and cobras (*Naja* spp.) appeared to be related to a recent ancestral protein that shared a common node with SNTX from the sea kraits (*Laticauda* spp.). Within the sea snake SNTX, Lh_FTX001 from *H. curtus* (Penang) is closely related to the more basal SNTX of *H. schistosus* and the further derived SN160 (*H. curtus*, Beihai) and Short Neurotoxin 1 (*H. cyanocinctus*) but the branch lengths were short and this implied little genetic differences. On the other hand, the LNTX sequences of Lh_FTX013 (Penang) and A3FM53 from *H. curtus* were identical, while there is no LNTX sequence of *H. schistosus* available for comparison. 

The close phylogenetic relationship among the SNTX and LNTX of sea snakes, sea kraits and cobras support the wide cross-reactivity of Sea Snake Antivenom (SSAV) [33,34], which is the only specific antivenom indicated for the treatment of sea snake envenomation. SSAV is raised against the beaked sea snake (*H. schistosus*, Penang) specifically but studies have extensively demonstrated that it could effectively cross-neutralize the toxicity of most other marine elapids of various *Hydrophis* spp. (including *H. platurus* and its most important neurotoxin), sea kraits (*Laticauda* spp.) and related principal toxicity [13,14,32,33]. The extensive cross-reactivity of SSAV is indicative of substantially conserved antigenicity in the SNTX and LNTX, respectively. The antigenicity of SNTX and LNTX, however, may possibly vary in view of the more variable amino acid compositions and the further relatedness between the two toxin groups. 

### 2.5. Clinical Relevance and Antigencity of Three-Finger Toxins

**Figure 5:**
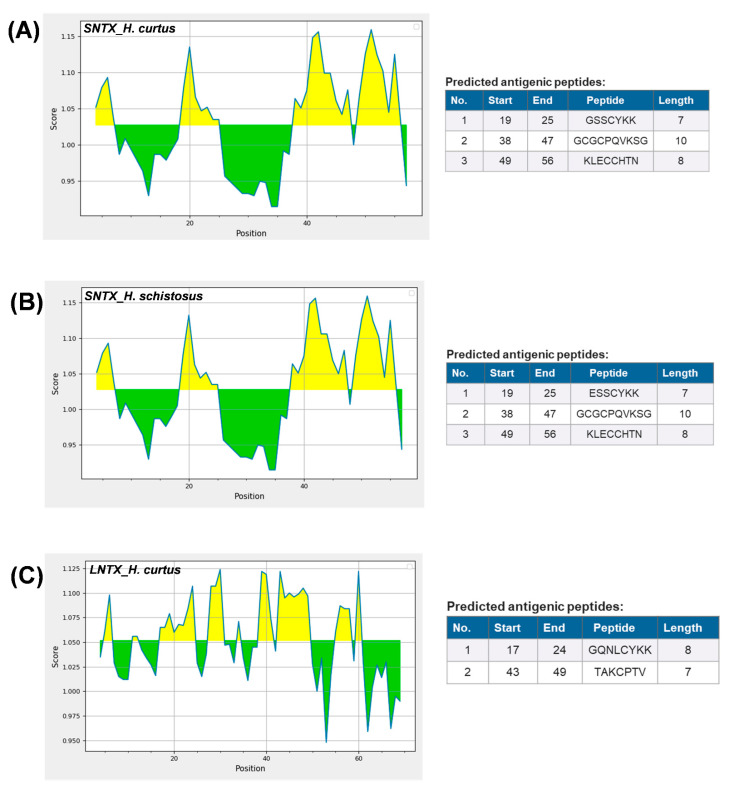
Predicted antigenicity of alpha-neurotoxin proteins from *Hydrophis curtus* and *Hydrophis schistosus* with Kolaskar and Tongaonkar method [45]. (**A**) Short neurotoxin, *H. curtus*, accession: Lh_FTX001. (**B**) Short neurotoxin, *H. schistosus*, accession: P68415. (**C**) Long neurotoxin, *H. curtus*, Lh_FTX013. Yellow areas corresponded to antigenicity score above threshold (1.024) proposed to be part of B-cell epitope.

The dominant 3FTX expressed, Lh-FTX13 and Lh_FTX01, were corresponding to the major SNTX and LNTX reported in the venom proteome of *H. curtus* [13]. Both the SNTX and LNTX of *H. curtus* are highly lethal (LD_50_ = 0.10 µg/g and 0.24 µg/g, respectively) and contributing to the neurotoxicity and lethality of the venom (LD_50_ = 0.20 µg/g). In the current transcriptomic study, Lh_FTX001 (SNTX) has a higher relative abundance compared to Lh_FTX013 (LNTX), in agreement with the proteome reported in which SNTX was more abundantly present than LNTX in the venom. The SNTX-predominating venom phenotype is common in several other sea snake species besides *H. curtus* (Penang), including the congeneric *H. schistosus* [36], *H. platura* [14], *H. cyanocinctus* [16] and the paraphyletic *Aispyrus laevus* [37]. The ecological role of SNTX and LNTX in the venom is associated with predatory function, whereby the venom composition is streamlined to incapacitate the fast-moving teleost-based prey (fishes). In envenomation, these are the toxins that block post-junctional nicotinic receptors, resulting in neuromuscular paralysis, respiratory failure and death [38]. Ergo, the treatment outcome of envenomation is principally determined by the antivenom efficacy in neutralizing the principal toxins of the venom. It has been shown that SNTX are more reversible than LNTX in the binding of nicotinic receptors (nAChR), notwithstanding the fact that they are less effectively neutralized by antivenoms [32,34,39,40]. From the immunological perspective, it is possible that LNTX and SNTX vary in their antigenicity, hence the discrepancy in immunorecognition and efficacy of antivenom. Figure 5 shows the predicted antigenicity of alpha-neurotoxin proteins from *H. curtus* (SNTX and LNTX), *H. schistosus* (SNTX) and *L. colubrina* (LNTX). SNTX of *H. curtus* and *H. schistosus* have, respectively, 3 prominent epitopes with antigenicity scores beyond 1.10. All three antigenic peptide segments of the two *Hydrophis* sea snakes comprise residues across 19–25, 38–47 and 49–56, with each antigenic pair sharing highly conserved amino acid residues. The epitope prediction suggested that SNTX of *H. schistosus*, the species whose venom is used in raising Sea Snake Antivenom, is antigenic to produce antibodies that should be equally effective in cross-neutralizing the SNTX of *H. curtus*. This is in line with the reported neutralization potency of Sea Snake Antivenom against the neurotoxins of *H. schistosus* and *H. curtus* at 0.35 mg/mL and 0.34 mg/mL, respectively. On the other hand, the LNTX of *H. curtus* exhibited two epitopes (residues 17–24 and 43–49), while the LNTX sequence of *H. schistosus* is not available from the database for comparison. We predicted that the LNTX of *H. schistosus* should share similar epitopes with the LNTX of *H. curtus*, since the Sea Snake Antivenom could effectively cross-neutralize the *H. curtus* LNTX (potency = 0.78 mg/mL), albeit less potent than it was against that of *H. schistosus* (potency = 1.38 mg/mL) [13,34]. Interestingly, the SNTX and LNTX do not seem to share much common epitopes, implying limited synergistic cross-reactivity that can be resulted from one antibody toward both toxins. Furthermore, the neutralization potency of antivenom against SNTX is generally lower than LNTX, despite the presence of prominent epitopes in the SNTX protein. Hence, antivenom manufacturers should ensure that the product contains adequate antibodies that are sufficiently immunoreactive toward both types of neurotoxins, so that the reversal of neurotoxicity caused by either SNTX or LNTX can be effective. The production of antivenom toward specific toxin targets can be improved through recent innovations of recombinant technologies [41,42] and re-formulation of specific toxin-targeting antivenom [43,44] to achieve higher potency against the different toxins.

### 2.6. Phospholipases A_2_

**Figure 6:**
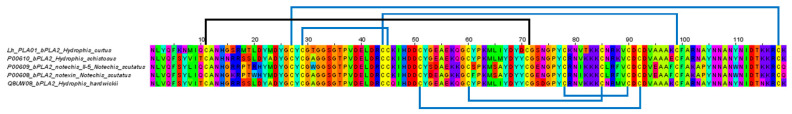
Multiple sequence alignment of the major phospholipase A_2_ (PLA_2_, Lh_PLA01) of *Hydrophis curtus* (Penang) and related sequences. The PLA_2_ shared highly conserved cysteine residues and seven disulfide bridges. Blue lines: conservative disulfide bonds; black lines: additional disulfide bond for Group IA PLA_2_.

Phospholipase A_2_ transcripts represented the second most abundantly expressed toxin genes in *H. curtus* venom-gland transcriptome. The major transcript coding for PLA_2_, that is, Lh_PLA01 contains a full sequence of 118 amino acid residues and was annotated to the basic PLA_2_ 73 (Q8UW30) from Hardwick’s sea snake (of unknown locale, possibly from southern China), based on 92% sequence similarity. Lh_PLA01 belongs to Group IA PLA_2_ and is a D49 subtype of snake venom PLA_2_. It has a conserved Ca^2+^ binding loop that lies between residues 25 and 33 (consensus sequence: Y25-G-C-Y/F-C-G-X-G-G33) and His48 as well as Asp49 which are critical for enzymatic activity [46] (Figure 6). High sequence similarity was also observed when comparing Lh_PLA01 with the basic PLA_2_ of *H. schistosus* (P00610) (Figure 6), a highly lethal myotoxin that causes systemic myotoxicity and renal failure secondary to rhabdomyolysis [47]. Unlike the myotoxic PLA_2_ of *H. schistosus*, the major enzymatic PLA_2_ of *H. curtus* was found to be non-lethal in mice [13,36]. The finding implied that Lh_PLA01 has a variable sequence that probably does not contribute to toxicity, or, it requires the presence of subunit to form PLA_2_ complex in order to produce toxic activity. Lind and Eaker [48] pointed out that in toxic elapid PLA_2_s that act in monomeric form, such as the myotoxin from *H. schcistosus*, notexin and notechis-II5 (both are neurotoxic systemically while myotoxic locally) from *Notechis scutatus*, have a unique Lys-Lys-Lys sequence at positions 82-84 (Figure 6) not shared by beta-bungarotoxin PLA_2_ chain and most other non-myotoxic PLA_2_ variants. This sequence thus could be important for basic PLA_2_ to exert myotoxic and/or neurotoxic activity in monomeric form. Lh-PLA01 lacks this feature: the positively charged Lys82 was substituted with the neutral Thr82 (Figure 6) and the mutation probably has modified the characteristic cationic site crucial for the myotoxic activity of monomeric PLA_2_ [46]. More extensive sequence comparison in conjunction with chemical modification studies should clarify the phenomenon. 

In the present study, the transcript expression level of PLA_2_ (~20% of total toxin FPKM) was lower than the protein abundance of PLA_2_ reported in proteomics (50–70% of the total venom proteins) [12,13]. The discrepancy could be due to the fact that the mRNAs of various toxins were synthesized at different rates over days and weeks, while the venom-gland tissue was harvested at a certain time point, typically a few days after venom milking. Moreover, it is reasonable to think that the diverse mRNAs had varying half-lives and were subjected to complex regulation processes like post-transcriptional and post-translational modifications [49] which further modulated the maturation of the proteins in the final venom product. The lack of correlation between venom gene expression levels and protein abundances has also been observed in several previous studies [22,23,24,25,50], presumably due to the reason(s) above.

## 3. Conclusions

The venom-gland transcriptome of *H. curtus* from the Peninsula of Malaysia was *de novo* assembled, unveiling the diversity of venom genes in this species. Three-finger toxins constituted the major genes expressed in the venom glands, with SNTX and LNTX being the most abundant, consistent with their role as the principal toxins implicated in the pathophysiology of snakebite envenomation. The findings enriched the toxin knowledgebase of sea snakes and shed light on the medical importance of the venom. 

## 4. Materials and Methods

### 4.1. Preparation of Snake Venom-Gland Tissue

The sea snake, *H. curtus* was an adult specimen from the northern waters of Penang Island west of Peninsular Malaysia. The venom was milked four days prior to venom gland tissue collection to promote transcription [51]. The venom glands were collected following euthanasia and sectioned into dimensions of 5 × 5 mm. The sectioned tissue was immersed in RNAlater^®^ solution (Ambion, TX, USA) at 4 °C overnight and stored at −80 °C until further use. The study was carried out in line with protocols approved by the Institutional Animal Use and Care Committee (IACUC) of University of Malaya, Malaysia (Approval code: #2013-11-12/PHAR/R/TCH).

### 4.2. RNA Extraction and Purification

The venom-gland tissue was homogenized in a 1 mL glass homogenizer with TRIzol solution (Invitrogen, Carlsbad, CA, USA). This was followed by the isolation using chloroform and treated with RNA-free DNAase I (Thermo Fisher Scientific, Waltham, MA, USA), to separate cellular debris and residual DNA. The isolated RNA was then purified via isopropyl alcohol ethanol precipitation. Polyadenylated mRNA was subsequently purified with oligo(dT) magnetic beads (Illumina TruSeq Stranded mRNA) (Illumina, San Diego, CA, USA) as per manufacturer’s instructions. The quality of the purified total RNA was assessed using Agilent 2100 Bioanalyzer (RNA 6000 NanoKit) (Agilent Technologies, Waldbronn, Germany).

Enriched poly(A)^+^ mRNA isolated from the total venom-gland RNA was used for cDNA construction. The isolated mRNA was fragmented into short fragments, which acted as templates for cDNA synthesis [52]. Random hexamer-primer (*N6*) was used to synthesis the first-stranded cDNA, followed by second-strand cDNA synthesis with the double-stranded cDNA as input materials, using second strand buffers, dNTPs, RNase H and DNA polymerase I. From these cDNA, a paired-end library was synthesized using the Genomic Sample Prep kit (Illumina, San Diego, CA, USA), according to the manufacturer’s instructions. The cDNA fragments generated were purified with QIAquick PCR extraction kit (Qiagen, Valencia, CA, USA) and dissolved in elution buffer for end repair and the addition of poly(A) to aid in the subsequent ligation of Illumina adaptors that contain a single thymine (T) base overhang at the 3′ ends. Following the ligation, these cDNA fragments were amplified via polymerase chain reaction (PCR) electrophoresed on a 1.5–2% TAE (Tris base, acetic acid and EDTA) agarose gel. From the gel, suitable fragments (200–700 bp) were selected as templates for subsequent PCR amplification. Sequencing of the amplified samples library was achieved in a single lane on the Illumina HiSeq™ 2000 platform (Illumina, San Diego, CA, USA)) with 100-base-pair, paired-end reads.

### 4.3. Filtration of Raw Sequenced Reads

Sequenced data generated from Illumina HiSeq™ 2000 platform were transformed by base calling into sequence data, called the raw reads and stored in a FASTQ format. Prior to transcriptome assembly, raw reads were filtered to generate clean reads as part of the quality control process in the pre-analysis stage [53]. This involved the removal of (i) adaptors; (ii) reads with >5% of unknown nucleotides or (iii) low-quality reads with >20% of low-quality bases (determined as base quality < 10). 

### 4.4. De Novo Transcriptome Assembly

The *de novo* transcriptome assembly was performed using a short-reads assembly program, Trinity (version 2.0.6) [54,55]. Three independent software modules, that is, Inchworm, Chrysalis and Butterfly, comprised the Trinity program were sequentially applied to process the large volumes of RNA-seq reads. In brief, this was based on the algorithm of *de Bruijn* graphs construction, which began by aligning *k*-mers (*k* = 25) and reads with a certain length of overlap were joined to form linear contigs. The reads were mapped back onto contigs and by referring to paired-end reads, contigs from the same transcript, as well as the distances between them were determined. The contigs were then partitioned into clusters, each of which carried a complete set of *de Bruijn* graphs (representing the transcriptional complexity at a given gene or locus). The graphs were independently processed to obtained full-length transcripts for alternatively spliced isoforms and to tease apart transcripts that corresponded to paralogous genes. The clean read Q20 percentage, a point of reference for quality control assessment was obtained as a benchmark for successful *de novo* assembly of the transcriptome. 

### 4.5. Clustering and Functional Annotation of Transcripts

The transcript sequences generated through Trinity were called Unigenes. Unigenes from the transcriptome assembly were further processed for sequence splicing and redundancy removal with TGI clustering tools (TGICL, version 2.1) to acquire non-redundant (NR) transcripts at the longest possible length [56]. The transcripts were then subjected to family clustering, which resulted in two classes of transcripts: (a) clusters, with a prefix CL and the cluster ID behind as contig; (b) singletons, whose ID was simply left with a prefix of Unigene. In each cluster, there were several transcripts with sequence similarities among them being >70%; while the singletons ‘Unigenes’ lack overlapping with other fragments at the given stringency. The value 70% was used to categorize the assembled sequences based on similarity; sequences similar to each other (may or may not be homologous as having >90% similarity) were grouped under a cluster comprising various contigs. 

Following this, transcript Unigenes were then aligned with BLASTx to protein database in priority order to NCBI non-redundant database (NR), with a cut-off value of E < 10^−5^. Proteins with the highest ranks in the BLASTx results were referred to determine the coding region sequences of Unigenes, followed by translation into amino acid sequences (using standard codon table). Hence, both nucleotide sequences (5′ to 3′) and amino acid sequences of the Unigene-coding regions was acquired. To remove redundancy from each cluster, the longest sequence in each cluster was chosen as the transcript, meanwhile, the length of scaffold was extended based on overlapping sequences using Phrap assembler (release 23.0) (http://www.phrap.org). The distributions of the length of contigs, scaffolds and Unigenes were calculated and the N50 length (assembly quality indicator) was set at N50 > 500 for assembly success.

### 4.6. Quantifying Transcript Abundance

Clean reads were aligned to Unigene using Bowtie2 [57]. The transcript abundances were calculated using RNA-seq with expectation maximization (RSEM) tool [58].

Fragments per kilobase of exon model per million reads mapped (FPKM) were used to determine the transcript abundance for the identified genes [59]. FPKM is the summation of normalized read counts based on gene length and the total number of mapped reads. The data was obtained using RSEM tool in conjunction with Trinity based on a computational formula:

$$FPKM\;of\;gene\;A\;=\frac{10^{6}B}{NC∕1000}$$

FPKM is the expression of gene *A*; *B* is the number of fragments/reads which are aligned to gene *A*; *N* is the total number of fragments/reads that are aligned to all genes; *C* is the base number in the coding sequence of gene *A*.

### 4.7. Categorization of Transcripts

The *de novo* assembled transcripts were subjected to BLASTx search to obtain the closest resembling sequences from the NR protein database for further classification based on functional annotations. The transcripts (Unigenes) were then sifted to remove those with an FPKM value of less than 1, followed by categorization into three groups: “toxins,” “non-toxins” and “unidentified” [21,23]. “Toxin” transcripts were recruited by toxin-related keyword searches against the annotated transcripts. “Non-toxin” and “unidentified” groups contain transcripts of cellular proteins or house-keeping genes and transcripts that could not be identified, respectively. The redundancy of gene expression was determined by dividing the total FPKM of each group by the total number of transcripts in the respective group of transcripts [21]. In the toxin group, the amino acid sequences were used to further validate the toxin identity through BLASTp suite (Basic Local Alignment Search Tool-Protein) in the UniProt (Universal Protein Resource Knowledgebase) database platform. The transcripts were searched against Serpentes database (taxid: 8570) and validated based on the lowest E-score value with the highest percentage of sequence similarity (updated as of 29 June 2020). 

### 4.8. Multiple Sequence Alignment

Multiple sequence alignment was conducted using Jalview software (version 2.11.1.0) [60] and MUSCLE (Multiple Sequence Comparison by Log-Expectation) [61] program. Sequences of related species used in multiple sequence alignment were retrieved from UniProtKB depository (accessed date: 14 September 2020) (http://www.uniprot.org/). The selection was based on their relevance to the toxins in comparison to elucidate the similarity and variation as well as conserved regions of the sequences.

### 4.9. Phylogenetic Analysis

Sequences of long and short neurotoxins annotated for *H. curtus* of Penang (this work) and representative species of sea snakes, sea kraits and cobras (retrieved from Universal Protein Knowledgebase, UniProtKB, http://www.uniprot.org/) (accessed date: 14 September 2020) were used to construct the phylogenetic tree. The tree was constructed with Molecular Evolutionary Genetics Analysis (MEGA) Version X [35] applying the Dayhoff (PAM) substitution model (+G) [62]. Bootstrap test (1000 replicates) was computed for the confidence limits of the constructed phylogenetic tree [63].

### 4.10. Scale-Based B-Cell Epitope Prediction

The antigenic determinants (epitopes) of toxins were predicted using a scale-based B-cell epitope prediction software applying Kolaskar and Tongaonkar antigenicity prediction algorithm (http://tools.immuneepitope.org) (accessed on 27 November 2020) [45]. Default parameters and window size 7 were used in the analysis for predicting potentially antigenic regions of amino acids in the sequences

### 4.11. Supporting Data

Sequencing data from the *de novo* venom-gland transcriptomics of *H. curtus* was deposited in National Centre for Biotechnology Information (NCBI) Sequence Read Archive (https://submit.ncbi.nlm.nih.gov/subs/sra/) (submitted on 29 December 2020) under SRA accession: PRJNA688573.

## Figures and Tables

**Table 1:** Overview of the output statistics. The sequencing and assembly quality of the venom gland transcriptome of *Hydrophis curtus*.

Parameter	Output Statistics
Total raw reads	57,606,566
Total clean reads	54,140,326
Total clean nucleotides (nt)	4,872,629,340
Q20 percentage	98.60%
*N* percentage	<0.01%
GC percentage	44.63%
**Contigs created**	**126,790**
Total length (nt)	51,459,117
Mean length (nt)	406
N50	921
**Unigenes/transcripts assembled**	**82,209**
Total length (nt)	69,679,280
Mean length (nt)	848
N50	2073
**Unigene/transcripts assembled (FPKM > 1)**	**70,564**
Unidentified	45,616 (17.97%)
-Redundancy (FPKM abundance/number of transcripts)	3.8
Non-toxin	24,852 (33.86%)
-Redundancy (FPKM abundance/number of transcripts)	13.16
Toxin	96 (48.18%)
-Redundancy (FPKM abundance/number of transcripts)	4847.54

Q20 percentage is the proportion of nucleotides with a quality value larger than 20; *N* percentage is the proportion of unknown nucleotides in clean reads; GC percentage is the proportion of guanidine and cytosine nucleotides among total nucleotides; N50 is the shortest contig length needed to cover 50% of the transcriptome; FPKM stands for Fragments Per Kilobase of transcript per Million mapped reads; Redundancy refers to the abundance of expression per gene transcript.

**Table 2:** Overview of toxin genes in venom-gland transcriptome of Malaysian *Hydrophis curtus*.

Toxin Family/ID	UniProt Accession Code	*Species*	Expression Abundance (%)
**Three-Finger Toxin (3FTx)**			**77.26**
**S-3FTX**			**56.48**
Short neurotoxin SN160	Q8UW27	*H. hardwickii*	56.43
Short neurotoxin homolog NTL4	Q9YGI8	*B. multicinctus*	<0.01
Short neurotoxin OH-35	Q53B49	*O. hannah*	<0.01
3FTx	C6JUP5	*M. corallinus*	<0.01
putative three-finger toxin precursor	F5CPD1	*M. altirostris*	<0.01
Short neurotoxin OH-26	Q53B52	*O. hannah*	<0.01
Cytotoxin homolog 5V	Q9W716	*Naja atra*	0.02
Cytotoxin homolog 5	Q91137	*Naja atra*	0.01
Cytotoxin A5	P62375	*Naja atra*	0.01
Cardiotoxin-like protein BMLCL	Q9PW19	*B. multicinctus*	<0.01
**L-3FTX**			**20.78**
Long neurotoxin 2	A3FM53	*H. hardwickii*	20.74
Alpha-bungarotoxin isoform A31	P60615	*B. multicinctus*	0.02
Kappa-bungarotoxin	P01398	*B. multicinctus*	0.01
Long neurotoxin homolog NTL2	Q9YGH9	*B. multicinctus*	0.01
Long chain neurotoxin 6	U3FYQ0	*M. fulvius*	<0.01
Neurotoxin BM10-1-like	Q70WS8	*B. multicinctus*	<0.01
Long chain neurotoxin 2	U3FAC0	*M. fulvius*	<0.01
**NC-3FTX**			**<0.01**
Weak toxin 1	Q8AY51	*B. candidus*	<0.01
**Phospholipase A_2_**			**18.88**
Basic phospholipase A_2_ 73	Q8UW30	*H. hardwickii*	18.84
Acidic phospholipase A_2_	P00606	*B. multicinctus*	0.03
Phospholipase A_2_ MALT0035C	F5CPF1	*M. altirostris*	0.01
Phospholipase A_2_ GL16-1	Q8JFB2	*L. semifasciata*	<0.01
Basic phospholipase A_2_ beta-bungarotoxin A1 chain	P00617	*B. multicinctus*	<0.01
Phospholipase A_2_ pkP2	Q8JFG2	*L. semifasciata*	<0.01
**Cysteine-rich Secretory Protein**			**3.34**
Cysteine-rich venom protein 2	Q8UW11	*H. hardwickii*	3.34
Cysteine-rich secretory protein Bc-CRPb	F2Q6G2	*B. candidus*	0.01
**Phospholipase A_2_ Inhibitor**			**0.13**
phospholipase A2 inhibitor-like	A0A6J1W4V4	*N. scutatus*	0.13
**C-type Lectin**			**0.12**
C-type lectin 1	A3FM55	*H. hardwickii*	0.07
C-type lectin isoform 1	H8PG89	*P. nigriceps*	0.04
Venom C-type lectin mannose binding isoform 4	D2YVK4	*H. stephensii*	0.01
**Kunitz-type Protease Inhibitor**			**0.09**
Putative Kunitz-type serine protease inhibitor	B2BS84	*A. labialis*	0.06
Kunitz-type protease inhibitor 1	V8N7R6	*O. hannah*	0.01
Kunitz-type serine protease inhibitor homolog beta-bungarotoxin B1 chain	Q8AY46	*B. candidus*	0.01
Kunitz-type serine protease inhibitor PILP-2	B4ESA3	*B. multicinctus*	0.01
Kunitz-type serine protease inhibitor spermatin	C1IC52	*W. aegyptia*	<0.01
Kunitz-type serine protease inhibitor 28	F8J2F3	*D. coronoides*	<0.01
Protease inhibitor 4	C1IC53	*W. aegyptia*	<0.01
Kunitz-type serine protease inhibitor vestiginin-2	A6MFL2	*D. vestigiata*	<0.01
Kunitz-type serine protease inhibitor	P20229	*Naja naja*	<0.01
Kunitz-type serine protease inhibitor 161	F8J2F4	*D. coronoides*	<0.01
**Snake Venom Metalloproteinase**			**0.08**
Zinc metalloproteinase-disintegrin-like NaMP	A8QL59	*N. atra*	0.05
Porphyriacase-1	B5KFV2	*P. porphyriacus*	0.01
Scutatease-1	B5KFV7	*N. scutatus*	0.01
Zinc metalloproteinase-disintegrin-like BmMP	A8QL49	*B. fasciatus*	<0.01
Zinc metalloproteinase-disintegrin-like MTP9	F8RKV9	*D. coronoides*	<0.01
Carinatease-1	B5KFV1	*T. carinatus*	<0.01
Snake venom metalloproteinase-disintegrin-like mocarhagin	Q10749	*N. mossambica*	<0.01
Zinc metalloproteinase-disintegrin-like BfMP	A8QL48	*B. fasciatus*	<0.01
Zinc metalloproteinase-disintegrin-like NaMP	A8QL59	*N. atra*	<0.01
Stephensease-1	B5KFV4	*H. stephensii*	<0.01
**Cystatin**			**0.06**
Cystatin	E3P6N8	*P. australis*	0.03
Cystatin	V8NX38	*O. hannah*	0.02
Cystatin-B	V8P5H9	*O. hannah*	0.01
**Dipeptidyl Peptidase IV**			**0.02**
Venom dipeptidylpeptidase IV	A6MJI1	*T. carinatus*	0.02
Snake Venom Serine Protease			0.01
Serine protease harobin	Q5MCS0	*H. hardwickii*	0.01
**5’ Nucleotidase**			**<0.01**
5’ nucleotidase	A6MFL8	*D. vestigiata*	<0.01
5’-nucleotidase domain-containing protein 3	V8P4R1	*O. hannah*	<0.01
5’-nucleotidase	V8NYW9	*O. hannah*	<0.01
**Vascular Endothelial Growth Factor**			**<0.01**
Vascular endothelial growth factor C	V8NCP7	*O. hannah*	<0.01
**Hyaluronidase**			**<0.01**
Hyaluronidase	V8PHI0	*O. hannah*	<0.01
Hyaluronidase	V8PFK9	*O. hannah*	<0.01
Hyaluronidase	V8P1Z9	*O. hannah*	<0.01
**Phosphodiesterase**			**<0.01**
2’,5’-phosphodiesterase 12	V8PEM5	*O. hannah*	<0.01
**Waprin**			**<0.01**
Supwaprin-a	B5KGY9	*A. superbus*	<0.01
**Natriuretic Peptide**			**<0.01**
Natriuretic peptide Oh-NP	D9IX98	*O. hannah*	<0.01
Natriuretic peptide Na-NP	D9IX97	*N. atra*	<0.01
**Cobra Venom Factor**			**<0.01**
A.superbus venom factor 1	Q0ZZJ6	*A. superbus*	<0.01
**Nerve Growth Factor**			**<0.01**
NGF-Hop-5	R4G2H9	*H. bungaroides*	<0.01
Venom nerve growth factor 1	Q3HXY6	*N. scutatus*	<0.01
**Aminopeptidase**			**<0.01**
Aminopeptidase N	V8NGF6	*O. hannah*	<0.01
**Neprilysin**			**<0.01**
Neprilysin	V8NQ76	*O. hannah*	<0.01
**L-amino-acid Oxidase**			**<0.01**
L-amino-acid oxidase	A8QL51	*B. multicinctus*	<0.01
**Acetylcholinesterase**			**<0.01**
Acetylcholinesterase	Q92035	*B. fasciatus*	<0.01

Genus abbreviation: *A*, *Austrelaps*; *B*, *Bungarus*; *Demansia*/*Drysdalia*; *H*, *Hydrophis*/*Hoplocephalus*; *M*, *Micrurus*; *N*, *Naja/Notechis*; *O*, *Ophiophagus*; *P*, *Pseudechis*/*Parasuta*; *T*, *Tropidechis*; *W*, *Walterinnesia*.

**Table 3:** Full-length toxin transcripts derived from the venom-gland transcriptome of Malaysian *Hydrophis curtus*.

Transcript ID	Toxin Gene Family/Annotated ID	UniProt Accession Code	*Species*	Transcript Length (aa)	Annotated ID Length (aa)	Coverage	Coverage to Mature Chain (%)
	Three-Finger Toxin (3FTx)						
Lh_FTX01	Short neurotoxin SN160	Q8UW27	*H. hardwickii*	81	81	1–81	100
Lh_FTX02	Short neurotoxin homolog NTL4	Q9YGI8	*B. multicinctus*	71	86	16–86	100
Lh_FTX03	Short neurotoxin OH-35	Q53B49	*O. hannah*	63	86	15–85	100
Lh_FTX04	3FTx	C6JUP5	*M. corallinus*	62	79	15–78	98
Lh_FTX05	putative three finger toxin precursor	F5CPD1	*M. altirostris*	66	82	21–82	100
Lh_FTX06	Short neurotoxin OH-26	Q53B52	*O. hannah*	62	78	15–77	98
Lh_FTX08	Cytotoxin homolog 5V	Q9W716	*N. atra*	66	83	15–83	10
Lh_FTX10	Cytotoxin A5	P62375	*N. atra*	70	83	7–83	100
Lh_FTX11	Cytotoxin A5	P62375	*N. atra*	69	83	7–83	100
Lh_FTX12	Cardiotoxin-like protein BMLCL	Q9PW19	*B. multicinctus*	97	103	7–103	100
Lh_FTX13	Long neurotoxin 2	A3FM53	*H. hardwickii*	93	93	1–93	100
Lh_FTX14	Alpha-bungarotoxin isoform A31	P60615	*B. multicinctus*	77	95	15–91	95
Lh_FTX15	Kappa-bungarotoxin	P01398	*B. multicinctus*	72	87	15–86	94
Lh_FTX16	Long neurotoxin homolog NTL2	Q9YGH9	*B. multicinctus*	81	87	8–87	100
Lh_FTX17	Long chain neurotoxin 6	U3FYQ0	*M. fulvius*	72	84	14–84	100
Lh_FTX18	Neurotoxin BM10-1-like	Q70WS8	*B. multicinctus*	66	84	15–84	100
Lh_FTX19	Long chain neurotoxin 2	U3FAC0	*M. fulvius*	99	87	4–84	96
Lh_FTX20	Weak toxin 1	Q8AY51	*B. candidus*	70	86	17–86	100
	Phospholipase A_2_						
Lh_PLA01	Basic phospholipase A_2_ 73	Q8UW30	*H. hardwickii*	146	146	1–146	100
Lh_PLA02	Acidic phospholipase A_2_	P00606	*B. multicinctus*	132	145	14–145	100
	Cysteine-rich Secretory Protein						
Lh_CRP01	Cysteine-rich venom protein 2	Q8UW11	*H. hardwickii*	238	238	1–238	100
	C-type Lectin						
Lh_SCL01	C-type lectin 1	A3FM55	*H. hardwickii*	164	164	1–164	100
Lh_SCL02	C-type lectin isoform 1	H8PG89	*P. nigriceps*	172	157	1–157	100
Lh_SCL03	Venom C-type lectin mannose binding isoform 4	D2YVK4	*H. stephensii*	164	165	1–164	99
	Kunitz-type Serine Protease Inhibitor						
Lh_KUN01	Putative Kunitz-type serine protease inhibitor	B2BS84	*A. labialis*	249	252	1–252	100
Lh_KUN02	Kunitz-type protease inhibitor 1	V8N7R6	*O. hannah*	515	506	1–506	100
Lh_KUN03	Kunitz-type serine protease inhibitor homolog beta-bungarotoxin B1 chain	Q8AY46	*B. candidus*	86	85	1–84	98
Lh_KUN04	Kunitz-type serine protease inhibitor PILP-2	B4ESA3	*B. multicinctus*	66	83	1–82	98
Lh_KUN05	Kunitz-type serine protease inhibitor spermatin	C1IC52	*W. aegyptia*	79	81	1–79	98
Lh_KUN06	Kunitz-type serine protease inhibitor 28	F8J2F3	*D. coronoides*	66	83	18–83	100
Lh_KUN08	Kunitz-type serine protease inhibitor vestiginin-2	A6MFL2	*D. vestigiata*	71	83	16–81	97
Lh_KUN09	Kunitz-type serine protease inhibitor	P20229	*N. naja*	53	57	5–57	93
	Snake Venom Metalloproteinase						
Lh_SMP09	Carinatease-1	B5KFV1	*T. carinatus*	575	608	28–596	98
Lh_SMP10	Scutatease-1	B5KFV7	*N. scutatus*	586	608	28–608	100
Lh_SMP19	Zinc metalloproteinase-disintegrin-like NaMP	A8QL59	*N. atra*	590	621	28–618	98
	Cystatin						
Lh_CYS01	Cystatin	E3P6N8	*P. australis*	141	141	1–141	100
Lh_CYS02	Cystatin	V8NX38	*O. hannah*	164	171	8–171	96
	Dipeptidyl Peptidase IV						
Lh_DPP01	Venom dipeptidylpeptidase IV	A6MJI1	*T. carinatus*	753	753	1–753	100
	Snake Venom Serine Protease						
Lh_SSP01	Serine protease harobin	Q5MCS0	*H. hardwickii*	265	265	1–265	100
	5’ Nucleotidase						
Lh_NUC01	5’ nucleotidase	A6MFL8	*D. vestigiata*	559	559	1–559	100
	Vascular Endothelial Growth Factor						
Lh_VGF01	Vascular endothelial growth factor C	V8NCP7	*O. hannah*	421	421	1–421	100
	Hyaluronidase						
Lh_HYA01	Hyaluronidase	V8PHI0	*O. hannah*	481	469	19–469	96
	Waprin						
Lh_WAP01	Supwaprin-a	B5KGY9	*A. superbus*	64	75	16–75	100
	Cobra Venom Factor						
Lh_CVF01	A.superbus venom factor 1	Q0ZZJ6	*A. superbus*	1652	1652	1–1652	100
	Neprilysin						
Lh_NEP01	Neprilysin	V8NQ76	*O. hannah*	750	675	16–675	98

Genus abbreviation: *A*, *Austrelaps*; *B*, *Bungarus*; *Demansia*/*Drysdalia*; *H*, *Hydrophis*/*Hoplocephalus*; *M*, *Micrurus*; *N*, *Naja*/*Notechis*; *O*, *Ophiophagus*; *P*, *Pseudechis*/*Parasuta*; *T*, *Tropidechis*; *W*, *Walterinnesia*.

## Data Availability

Sequencing data from the *de novo* venom-gland transcriptomics of *H. curtus* was deposited in National Centre for Biotechnology Information (NCBI) Sequence Read Archive (https://submit.ncbi.nlm.nih.gov/subs/sra/) under SRA accession: PRJNA688573.

## Supplementary Materials

The following are available online at https://www.mdpi.com/2072-6651/13/2/127/s1, Table S1: Venom-gland transcriptomics of *Hydrophis curtus* (Penang): Toxin annotation and sequences.
